# Causal associations between human gut microbiota and hemorrhoidal disease: A two-sample Mendelian randomization study

**DOI:** 10.1097/MD.0000000000037599

**Published:** 2024-03-29

**Authors:** Fang Yang, Zhihua Lan, Huabing Chen, Rongfang He

**Affiliations:** a Anorectal Department of Traditional Chinese Medicine, The First Affiliated Hospital, Hengyang Medical School, University of South China, Hengyang, Hunan, China; b Department of Pathology, The First Affiliated Hospital, Hengyang Medical School, University of South China, Hengyang, Hunan, China.

**Keywords:** causal relationship, genetic analysis, gut microbiota, hemorrhoidal disease, mendelian randomization

## Abstract

Hemorrhoidal disease (HEM) is a common condition affecting a significant proportion of the population. However, the causal relationship between the gut microbiota and hemorrhoids remains unclear. In this study, we employed a Mendelian randomization (MR) approach to investigate the potential associations between them. In this study, the exposure factor was determined by selecting summary statistics data from a large-scale gut microbiome whole-genome association study conducted by the MiBioGen Consortium, which involved a sample size of 18,340 individuals. The disease outcome data consisted of 218,920 cases of HEM and 725,213 controls of European ancestry obtained from the European Bioinformatics Institute dataset. Two-sample MR analyses were performed to assess the causalities between gut microbiota and hemorrhoids using various methods, including inverse-variance weighting, MR-Egger regression, MR Pleiotropy Residual Sum and Outlier (MR-PRESSO), simple mode, and weighted median. Reverse MR analyses were performed to examine reverse causal association. Our findings suggest *phylum Cyanobacteria* (OR = 0.947, 95% CI: 0.915–0.980, *P* = 2.10 × 10 − 3), *genus Phascolarctobacterium* (OR = 0.960, 95% CI: 0.924–0.997, *P = *.034) and *family FamilyXI* (OR = 0.974, 95% CI: 0.952–0.997, *P* = .027) have potentially protective causal effects on the risk of HEM, while *genus Ruminococcaceae_UCG_002* (OR = 1.036, 95% CI: 1.001–1.071, *P* = .042), *family Peptostreptococcaceae* (OR = 1.042, 95% CI: 1.004–1.082, *P* = .029), *genus Oscillospira* (OR = 1.048, 95% CI: 1.005–1.091, *P* = .026), *family Alcaligenaceae* (OR = 1.048, 95% CI: 1.005–1.091, *P* = .036) and *order Burkholderiales* (OR = 1.074, 95% CI: 1.020–1.130, *P* = 6.50 × 10^−3^) have opposite effect. However, there was a reverse causal relationship between HEM and *genus Oscillospira* (OR = 1.140, 95% CI: 1.002–1.295, *P* = .046) This is the first MR study to explore the causalities between specific gut microbiota taxa and hemorrhoidal disease, which may offer valuable insights for future clinical interventions for hemorrhoidal disease.

## 1. Introduction

Hemorrhoids are swollen and inflamed blood vessels in the rectum and anus, respectively. Hemorrhoidal disease (HEM) occurs when hemorrhoids enlarge and cause symptoms.^[1]^ As a benign gastrointestinal condition, HEM is highly prevalent worldwide, particularly in industrial countries.^[2,3]^ Nearly 4 million Americans sought medical treatment for HEM in 2010.^[4]^ The annual economic burden of hemorrhoids in the population covered by employer insurance is estimated to be approximately $800 million, mainly because of the large number of hemorrhoidectomies.^[5]^ Surgery not only contributes to the medical burden but also brings about postoperative complications such as pain, bleeding, urinary retention, and perianal infection. However, if left untreated, HEM patients have an increased risk of colorectal adenomas or adenocarcinomas compared to normal individuals.^[6]^ Therefore, it is necessary to trace the underlying causes of hemorrhoids.

The exact cause of HEM is not fully understood, but several factors, including prolonged sitting or standing, chronic constipation or diarrhea, straining during bowel movements, obesity, pregnancy, a family history of hemorrhoids, and aging, can contribute to the development accompanied by weakened connective tissue support and degeneration of the hemorrhoidal plexus.^[7,8]^

Multiple studies have highlighted the significant role of the intestinal microbiome in preserving intestinal homeostasis.^[9]^ An imbalance in the gut microbiome can lead to benign intestinal diseases such as inflammatory bowel diseases and irritable bowel syndrome.^[10]^ Furthermore, the biodiversity of the gut microbiome of the rectum revealed significant differences between patients with anal fistulas and healthy individuals. For example, patients with anal fistulas showed enrichment of Synergistetes in their gut microbiome, whereas healthy individuals had a higher abundance of *Proteobacteria*.^[11]^ Alterations in the composition or diversity of the gut microbial community may influence the pathogenesis of hemorrhoids.^[12]^ However, there are currently no studies on the causal relationship between gut microbiome and HEM.

Due to the complex nature of the gut microbiome as an ecosystem, regulatory networks may exist among various types of bacteria and confounding factors that limit causal inferences between the gut microbiome and HEM. Mendelian randomization (MR) can be used to infer causal relationships between exposure factors and diseases through genetic variations. MR offers a convenient approach for investigating potential protective and risk factors for diseases and has been applied in several studies examining the relationship between the gut microbiome and gut diseases.^[13,14]^ In this analysis, a summary dataset from a genome-wide association study (GWAS) of the gut microbiome and HEM was utilized.

## 2. Method

### 2.1. Study design

**Figure 1.**
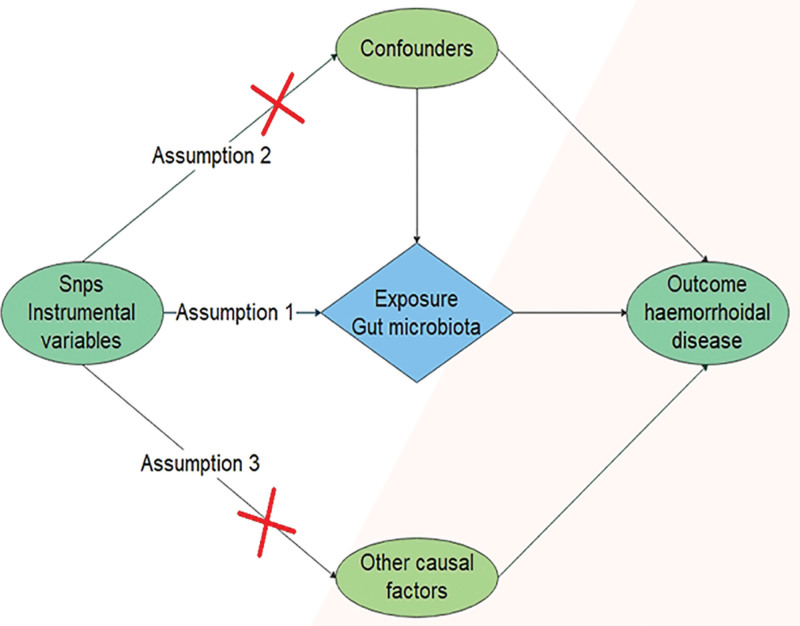
Three assumptions of Mendelian randomization.

Two-sample MR analyses were performed to investigate the causal effects of exposure (each bacterial taxon of gut microbiota) on the HEM, as presented in Figure 1, the analyses were performed alongside the essential assumptions: instrumental variables (IVs) were significantly associated with the gut microbiota, IVs were not related to any other confounding factors, and IVs only affected the outcome through gut microbiota.^[15]^

### 2.2. Data collection

The genetic IVs of each bacterial taxon were obtained from MiBioGen (link: https://mibiogen.gcc.rug.nl), which contains 16S ribosomal ribonucleic acid gene sequencing profiles and genotyping data from 18,340 individuals from the United States, United Kingdom, Finland, Sweden, Denmark, Netherlands, and other countries.^[16]^ A total of 9 phyla, 16 classes, 20 orders, 35 families, and 131 genera of bacteria associated with 122,110 variant sites were included in our study.

For HEM, single nucleotide polymorphism (SNP) information was obtained from the European Bioinformatics Institute (www.ebi.ac.uk/gwas) under accession number GCST90014033. The GWAS meta-analysis of HEM enrolled in this dataset showed 5 large population-based cohorts and included 218 920 HEM cases and 725 213 controls (n = 944133 in total), all of whom were of European ancestry.^[17]^ This research involves reanalyzing openly accessible data that has already been disseminated publicly, eliminating the necessity for ethical approval.

### 2.3. Filtration of genetic instrumental variables

First, a relaxed genome-wide significance threshold of *P* < 1 × 10^−5^ was employed, considering the limited number of SNPs available with a genome-wide significance level of *P* < 5 × 10 − 8.^[18]^ Second, SNPs with linkage disequilibrium r2 < 0.1 within a window of 500 kb were selected to ensure no linkage disequilibrium between the IVs.^[19]^ In cases where SNPs were absent in the outcome dataset, proxy-SNPs with linkage disequilibrium r2 > 0.8 were used as substitutes. Third, the F-statistic was calculated for each SNP, and SNPs with an F-statistic value below 10 were eliminated to mitigate the potential bias introduced by weak instruments (F = [R2 × (N−2)]/(1−R2), R2 = [2 × β^2^]/[2 × β2 + 2 × SE^2^ × N], where N is the sample size and β and SE represent the estimated effect size and standard error of the SNP on gut microbiota, respectively).^[20]^

### 2.4. Data analysis

**Figure 2.**
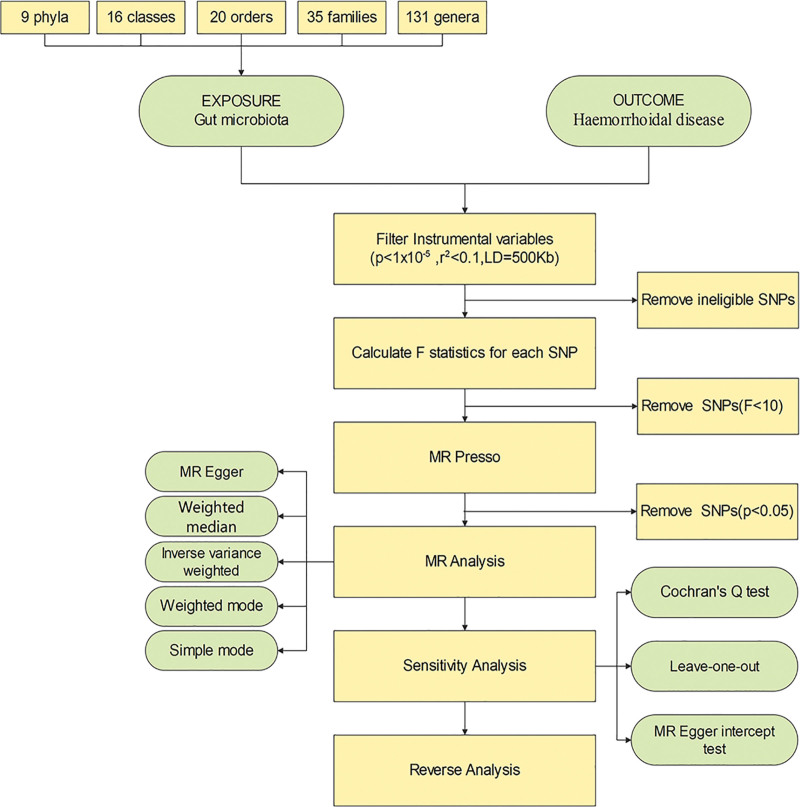
Workflow of the MR analysis. MR = Mendelian randomization.

The MR workflow was simply demonstrated in Figure 2. Inverse-variance weighted (IVW) as the main MR analysis method was used to evaluate the potential causal effects of each bacterial taxon on HEM risk.^[21]^ MR-Egger regression was used to assess horizontal pleiotropy. The MR-Egger intercept (*P* > .05) indicated that each SNP satisfied the Mendelian hypothesis.^[22]^ Mendelian Randomization Pleiotropy Residual Sum and Outlier (MR-PRESSO) analysis was conducted to identify and correct for the influence of heterogeneous outliers among the instruments. Heterogeneity was tested using the Cochrane Q test.^[21]^ The weighted median method was also used to validate the robustness of the IVW results. A leave-one-out sensitivity test was then employed to determine whether the exclusion of a single SNP had a significant impact on the inference of causal associations. Finally, reverse MR analysis was performed to examine whether a reverse causal association existed between HEM and the gut microbiota. In summary, we conducted MR and sensitivity analyses to ensure the reliability of GWAS data and obtain credible results.

The MR analysis was performed using the R package “TwoSampleMR.” All statistical analyses and data visualization were performed using R software 4.2.3.^[23]^

## 3. Result

**Figure 3.**
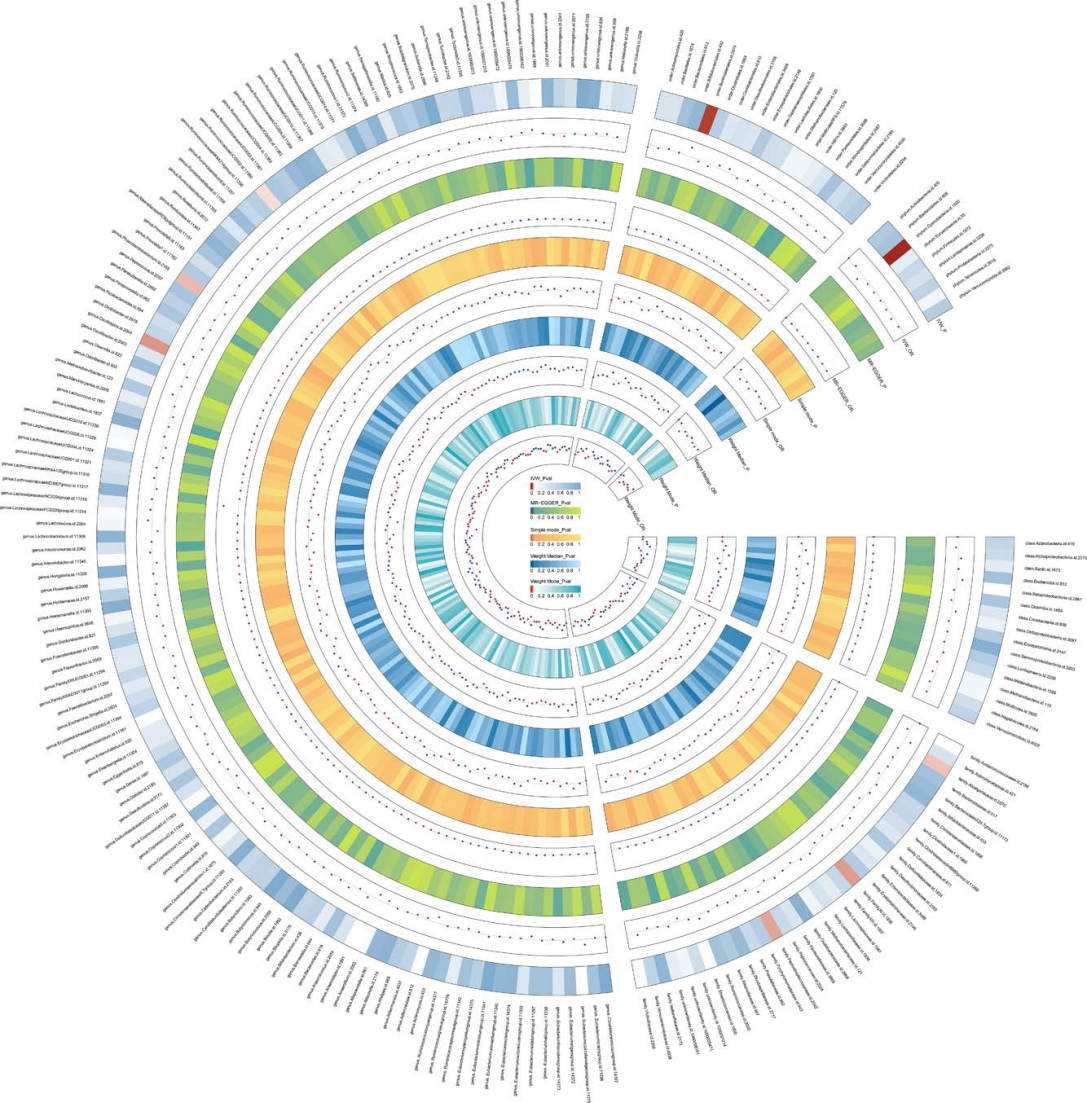
The circus plot presenting the 5 MR method results of all gut microbiota. MR = Mendelian randomization.

**Figure 4.**
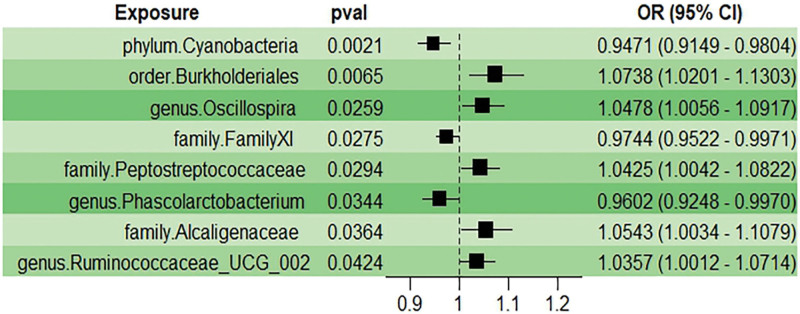
Forest plot showing significant results for the IVW analysis between the 8 gut microbial genera with the risks of HEM. HEM = hemorrhoidal disease, IVW = inverse-variance weighted.

A total of 2181 SNPs were screened as IVs from 211 gut microbiota samples (Table S1, Supplementary Digital Content, http://links.lww.com/MD/L988). Full results of the 5 MR methods between gut microbiota and HEM are shown in a circus plot (Fig. 3) and meticulously recorded in (Table S2, Supplementary Digital Content, http://links.lww.com/MD/L989. IVW analyses showed significant results in 1 phylum, 1 order, 3 families, and 3 genera(Fig. 4), including *phylum Cyanobacteria* (OR = 0.947, 95% CI: 0.915–0.980, *P* = 2.10 × 10 − 3), *genus Phascolarctobacterium* (OR = 0.960, 95% CI: 0.924–0.997, *P* = .034), *family FamilyXI* (OR = 0.974, 95% CI: 0.952–0.997, *P* = .027), *genus Ruminococcaceae_UCG_002* (OR = 1.036, 95% CI: 1.001–1.071, *P* = .042), *family Peptostreptococcaceae* (OR = 1.042, 95% CI: 1.004–1.082, *P* = .029), *genus Oscillospira*(OR = 1.048, 95% CI: 1.005–1.091, *P* = .026), *family Alcaligenaceae*(OR = 1.048, 95% CI: 1.005–1.091, *P* = .036), and *order Burkholderiales*(OR = 1.074, 95% CI: 1.020–1.130, *P* = 6.50 × 10 − 3). Eight gut microbiota taxa were associated with 90 SNPs and met the F-statistic >10 criteria.

**Figure 5.**
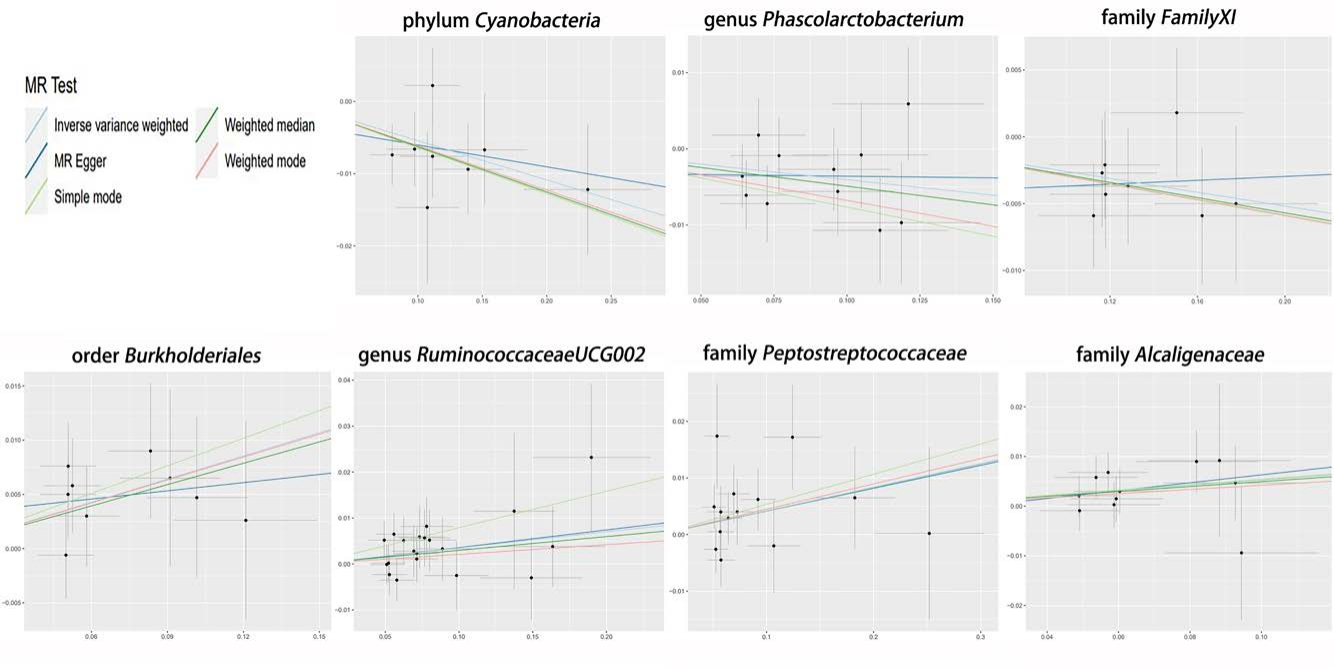
Scatter plots of the MR analysis. MR = Mendelian randomization.

**Figure 6.**
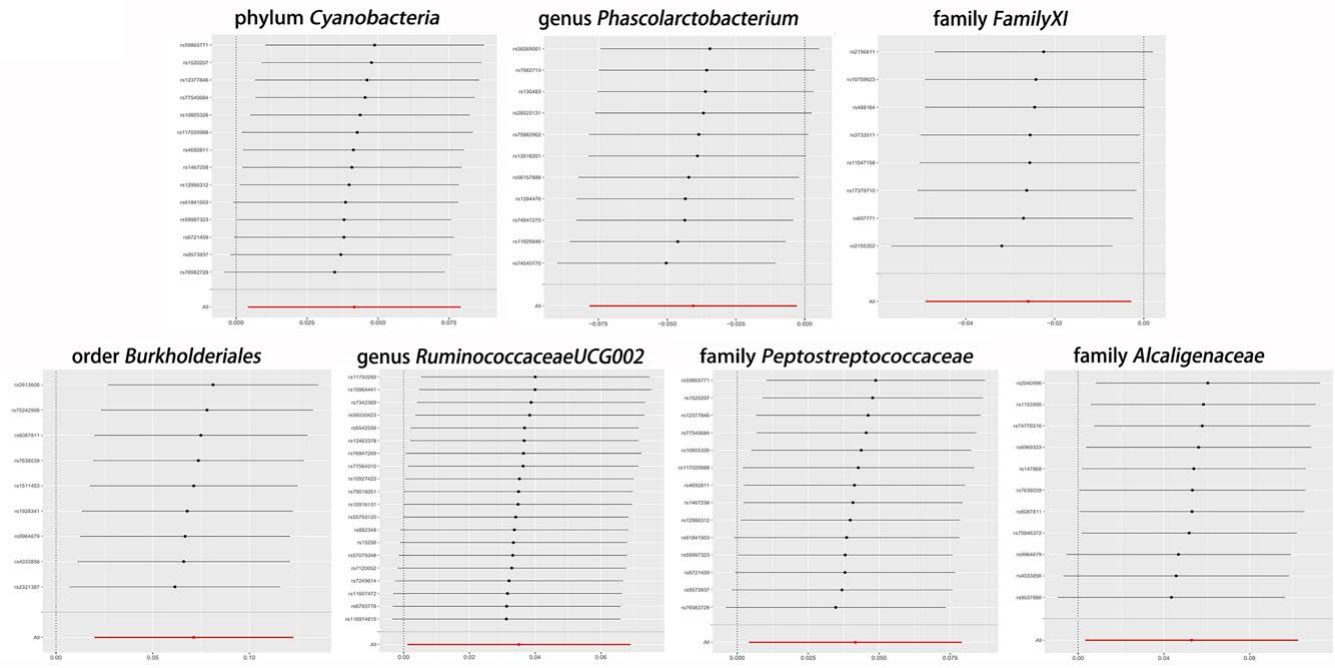
Leave-one-out results.

The sensitivity analysis results are presented in Table 1. Cochran’s test showed no significant heterogeneity among the gut microbiota variables, indicating that the IVs revealed no heterogeneity. MR-PRESSO analysis did not identify any outliers. The MR-Egger’s intercept analysis did not yield meaningful results, suggesting the absence of horizontal pleiotropy. The directions calculated using each method were consistent, except for the *family FamilyXI* (Fig. 5). The effect value calculated by the MR-Egger method for *family FamilyXI* was not consistent with the other 4 methods. However, the result was credible because there was no abnormal intercept or *P*-value of the MR-Egger test. The robustness of the results is demonstrated in Figure 6, where the outcomes of the leave-one-out technique remain consistent throughout the MR analysis. Regardless of the SNP removed, it had a minimal impact on the overall results.

**Table 1:** Sensitivity analysis of significant relationship between gut microbiota and HEM.

Gut microbitoa		Q_pval (IVW)	MR-PRESSO	Pleiotropy	
				Intercept	*P*-value
Phylum	*Cyanobacteria*	0.777	0.818	−3.014E−03	.683
Order	*Burkholderiales*	0.872	0.880	5.404E−04	.920
Genus	*Phascolarctobacterium*	0.796	0.810	−3.161E−03	.676
	*Ruminococcaceae_UCG_002*	0.483	0.489	−2.540E−03	.488
	*Oscillospira*	0.870	0.868	−2.898E−04	.973
Families	*FamilyXI*	0.943	0.957	−4.405E−03	.672
	*Peptostreptococcaceae*	0.614	0.671	8.914E−05	.980
	*Alcaligenaceae*	0.102	0.111	−1.275E−02	.076

HEM = hemorrhoidal disease, IVW = inverse-variance weighted, MR-PRESSO = Mendelian randomization pleiotropy residual sum and outliers, Q = cochran’s Q.

We performed reverse MR analysis using the IVW method to investigate the causal relationship between the 8 gut microbiota taxa and HEM. A total of 92 SNPs (*P* < 5 × 10−8) showing strong associations with HEM were selected as IVs.

As demonstrated in Table 2, *genus Oscillospira* showed a significant reverse causal link with HEM(OR = 1.140, 95% CI:1.002–1.295, *P* = .046), while no significant causal relationship was found between HEM and the other 7 gut microbiota taxa, including *phylum Cyanobacteria*(*P* = .390), *order Burkholderiales (P* = .487), *genus Phascolarctobacterium (P* = .562), *genus Ruminococcaceae_UCG_002 (P* = .982), *family FamilyXI (P* = .893), *genus Peptostreptococcaceae (P* = .450), and *family Alcaligenaceae (P* = .228), and the reliability of the findings was supported by MR-Egger regression analysis and Cochrane’s Q test.

**Table 2:** Sensitivity analysis of causal relationship between HEM and gut microbiota.

Gut microbiota (outcome)		SNP	SE	*P*-value (IVW)	OR (95% CI)	Pleiotropy		Heterogeneity
						Egger intercept	*P*-value	Q_pval (IVW)
Phylum	*Cyanobacteria*	76	0.070	.390	1.062 (0.9261–1.217)	−1.562E−02	.053	0.921
Order	*Burkholderiales*	76	0.047	.487	1.033 (0.9428–1.132)	−3.851E−03	.473	0.320
Genus	*Phascolarctobacterium*	76	0.060	.562	0.966 (0.8583–1.087)	−1.028E−02	.136	0.169
Genus	*Ruminococcaceae_UCG_002*	76	0.045	.982	0.999 (0.9147–1.091)	−1.489E-02	.005	0.544
Genus	*Oscillospira*	76	0.065	.046	1.140 (1.002–1.295)	−1.045E−02	.163	0.062
Family	*FamilyXI*	62	0.121	.893	1.016 (0.8018–1.288)	1.389E−02	.318	0.301
Family	*Peptostreptococcaceae*	76	0.048	.450	1.037 (0.9443–1.138)	7.419E−03	.174	0.359
Family	*Alcaligenaceae*	76	0.049	.228	1.061 (0.964–1.168)	−4.117E−03	.467	0.151

HEM = hemorrhoidal disease, IVW = inverse-variance weighted, OR = odds ratio, Q = cochran’s Q, SNP = single nucleotide polymorphism.

## 4. Discussion

This study represents the first MR investigation aimed at examining the causal relationship between the gut microbiota and HEM. These findings indicate that specific gut microbiota are causally associated with HEM.

Although the isolation and cultivation of *Cyanobacteria* in the human gut has not been achieved, genome analyses demonstrate that they harbor the genes for producing hydrogen gas, biosynthesizing vitamins, and generating energy, which suggests that they are beneficial to their host. Our study revealed that C*yanobacteria* serve as a protective factor, causally associated with HEM. Some previous studies showed *Cyanobacteria* members were more abundant in the infant gut with viral diarrhea and in adenoma mucosal biopsy samples.^[24,25]^ However, these results seem contradictory to the assumption that genome analyses are insufficient to imply causation.

*Phascolarctobacterium* was found to be a substantial acetate/propionate producer and positively correlated with the positive mood of humans.^[26,27]^ An integrated microbial-metabolomic analysis revealed a strong association between healthy twins and *Phascolarctobacterium faecium*, suggesting prospects for the development of live microbiome-modulating biotherapeutics.^[28]^ A previous MR analysis showed that *Phascolarctobacterium* promotes the absorption of serum vitamin D supplementation.^[29]^ After fecal microbiota transplantation, patients with slow-transit constipation have lower serum erucamide level, which is negatively correlated with *Phascolarctobacterium*.^[30]^

In patients with hepatic encephalopathy, the gut microbiota is characterized by an overgrowth of *Burkholderiales* and several other bacteria, capable of producing byproducts that may be neurotoxic,^[31]^ and these byproducts are also associated with epilepsy.^[32]^ It is still uncertain whether these metabolites affect neuromuscular motility, which is also a cause of HEM.^[17]^

MR and reverse MR analyses revealed a fascinating finding: *Oscillospira* and HEM emerged as mutually influential risk factors, underscoring the intricate nature of their relationship. A previous study has shown *Oscillospira* may play a role in aggravating constipation.^[33]^ Additionally, most studies demonstrated a significant association between constipation and HEM.^[34,35]^

The relative abundances of *Peptostreptococcaceae* and *Alcaligenaceae* were also higher in patients with constipation than in the normal group.^[36,37]^ An increased abundance of *Peptostreptococcaceae* was similarly associated with ulcerative colitis and may be related to the altered expression of antimicrobial peptides in the epithelium.^[38]^ Additionally, *Alcaligenaceae* has significant positive causal effects on weak muscle strength,^[39]^ which has been described as an important disorder in HEM.^[17]^

*Ruminococcaceae_UCG_002* appears to be a beneficial bacterium that produces short-chain fatty acids and mediates the metabolism of certain bile acids.^[40]^ However, *Ruminococcaceae_UCG_002* has been identified as a risk factor for Parkinson’s disease. The association of *Ruminococcaceae_UCG_002* under various conditions remains elusive.^[41]^

This study has several strengths. First, this is the first and largest comprehensive MR study of the gut microbiota at 5 levels, from genus to phylum and HEM. Second, the GWAS data utilized in our study to identify IVs exhibited a substantial sample size and improved accuracy. Third, the MR analysis results were unlikely to be biased by confounders, and reverse causation could be detected compared with conventional observational studies.

However, most of the GWAS data for gut microbiota and all GWAS data for HEM were from Europeans, which may have resulted in selection bias. Furthermore, summary statistics lacked grouping information for HEM, such as Goligher classification from I to IV^[42]^; therefore, we were unable to perform subgroup analyses.

## 5. Conclusion

A causal relationship was established between special gut microbiota and HEM through 2-sample MR. The potential mechanisms of these bacteria in neuromuscular motility and smooth muscle contraction deserve further study. These strains may also provide potential directions for the prevention of HEM.

## Acknowledgments

The authors would like to extend their gratitude to the participants and investigators involved in the GWAS analysis of hemorrhoidal disease. The authors would also like to thank the MiBioGen consortium for their generosity in sharing the genetic data.

## Author contributions

**Data curation:** Zhihua Lan.

**Supervision:** Rongfang He, Huabing Chen.

**Visualization:** Zhihua Lan.

**Writing – original draft:** Fang Yang.

**Writing – review & editing:** Fang Yang, Zhihua Lan, Rongfang He, Huabing Chen.

## Supplementary Material




